# Ibrutinib versus previous standard of care: an adjusted comparison in patients with relapsed/refractory chronic lymphocytic leukaemia

**DOI:** 10.1007/s00277-017-3061-3

**Published:** 2017-07-31

**Authors:** Lotta Hansson, Anna Asklid, Joris Diels, Sandra Eketorp-Sylvan, Johanna Repits, Frans Søltoft, Ulrich Jäger, Anders Österborg

**Affiliations:** 1 Department of Oncology-Pathology, Karolinska Institutet, Stockholm, Sweden; 2 Department of Hematology, Karolinska University Hospital, Stockholm, Sweden; 3 Department of Oncology, Karolinska University Hospital, Stockholm, Sweden; 4 Janssen EMEA, Beerse, Belgium; 5 Janssen Nordic, Stockholm, Sweden; 6 Medical University of Vienna, Vienna, Austria

**Keywords:** Ibrutinib, Relapsed refractory, Chronic lymphocytic leukaemia, Previous standard of care

## Abstract

This study explored the relative efficacy of ibrutinib versus previous standard-of-care treatments in relapsed/refractory patients with chronic lymphocytic leukaemia (CLL), using multivariate regression modelling to adjust for baseline prognostic factors. Individual patient data were collected from an observational Stockholm cohort of consecutive patients (*n* = 144) diagnosed with CLL between 2002 and 2013 who had received at least second-line treatment. Data were compared with results of the RESONATE clinical trial. A multivariate Cox proportional hazards regression model was used which estimated the hazard ratio (HR) of ibrutinib versus previous standard of care. The adjusted HR of ibrutinib versus the previous standard-of-care cohort was 0.15 (*p* < 0.0001) for progression-free survival (PFS) and 0.36 (*p* < 0.0001) for overall survival (OS). A similar difference was observed also when patients treated late in the period (2012-) were compared separately. Multivariate analysis showed that later line of therapy, male gender, older age and poor performance status were significant independent risk factors for worse PFS and OS. Our results suggest that PFS and OS with ibrutinib in the RESONATE study were significantly longer than with previous standard-of-care regimens used in second or later lines in routine healthcare. The approach used, which must be interpreted with caution, compares patient-level data from a clinical trial with outcomes observed in a daily clinical practice and may complement results from randomised trials or provide preliminary wider comparative information until phase 3 data exist.

## Introduction

Chronic lymphocytic leukaemia (CLL) is characterised by accumulation of malignant B lymphocytes in the lymph nodes, bone marrow and blood [1, 2]. CLL is the most common adult leukaemia in the developed world, with an annual incidence of 4.2 per 100,000, increasing to >30 per 100,000 among individuals over 80 years old [3]. In 2014, 15,720 diagnoses and 4600 deaths were reported in the USA and 18,480 cases were estimated to have been diagnosed in the EU5 in 2013 [4, 5]. As the average age of the global population increases, the incidence of CLL is expected to increase. In the USA, CLL diagnoses are estimated to increase by more than 50% by 2033 [6].

Although chemoimmunotherapy is effective as a first-line therapy in CLL patients without *TP53* dysfunction and long-term remissions after fludarabine/cyclophosphamide/rituximab (FCR) in IGHV-mutated patients may indicate a potential cure of some patients [7], CLL is normally considered incurable. Most CLL patients will eventually relapse from first-line treatment or become refractory to it [3, 4]. Until recently, available salvage regimens had limited efficacy in patients with a poor prognosis [8]. New molecular targets are being investigated in order to identify therapies to improve treatment outcomes in refractory CLL patients. Bruton’s tyrosine kinase (BTK) is a component of the B cell receptor (BCR) signalling pathway, which is critical in the maturation of B cells, and as such, BTK has emerged as a therapeutic target for B cell malignancies such as CLL [9].

Ibrutinib is a first-in-class inhibitor of BTK approved for the treatment of adult patients with previously untreated CLL. Ibrutinib as a single agent or in combination with bendamustine and rituximab (BR) is also approved for the treatment of adult patients with CLL who have received at least one prior therapy.

Ibrutinib monotherapy has been evaluated in a phase 3 study (RESONATE) in previously treated CLL patients against ofatumumab monotherapy [10]. The trial was a multicentre, open-label, phase 3 study, of 391 relapsed or refractory CLL patients receiving either ibrutinib orally at a dose of 420 mg daily until disease progression or standard dose of intravenous ofatumumab for up to 24 weeks. The RESONATE study demonstrated significant improvement with ibrutinib versus ofatumumab in progression-free survival (PFS) and overall survival (OS) in previously treated CLL patients. Long-term follow-up data for ibrutinib from a single-arm phase 2 study in treatment-naïve or previously treated CLL patients demonstrated a PFS rate of 69% and an OS rate of 79% at 2.5 years [11]. An additional phase 2 trial explored ibrutinib in a cohort of patients with del(17p)/*TP53* mutation with an ORR of 83% [12]. These data have been largely confirmed in two real-world setting studies performed in Sweden [13] and the UK/Ireland [14] but with significantly shorter PFS and OS among patients with del(17p) or *TP53* mutation in the Swedish study.

Health technology assessment bodies assessing new therapies require comparisons with a wide range of treatments. With the absence of direct head-to-head comparisons of single-agent ibrutinib with other widely used treatments in the previously treated CLL patient population, comparative evidence against previous standard of care in clinical practice can provide useful additional preliminary insights. However, naïve unadjusted comparisons of outcomes from different sources are prone to confounding bias due to lack of treatment non-randomisation and variation in prognostic factors between the treatment populations as well as being dependent on the generalizability of the control group.

The main objective of this study was to estimate the relative efficacy of ibrutinib versus previous standard-of-care treatments used in routine healthcare as used in the RESONATE trial in previously treated CLL patients. This estimate is based on a comparison of patient-level data from two different sources: the phase 3 RESONATE study and a retrospective, observational cohort of strictly consecutive patients from a well-defined geographical region [13]. By using this patient sample, this study aims to minimise these issues as it utilised a well-defined cohort of consecutive patients with almost complete follow-up from the Stockholm region of Sweden with absence of external referrals and controlled for baseline prognostic factors. Within the limitations that follow with such a study design, it can provide preliminary information on outcome with new versus previous therapies for previously treated CLL patients.

## Methods

### Study design

The study included two patient cohorts: an observational, historical, but strictly defined real-world cohort (subsequently referred to as the “Stockholm cohort”) [13] and a trial cohort from the RESONATE study (the RESONATE cohort) [11].

The Stockholm cohort included all CLL subjects treated with at least a second-line or subsequent therapy between 2002 and 2013 as identified from the Regional Cancer Registry in the Stockholm region (www.cancercentrum.se/stockholmgotland). These patients receive treatment and life-long follow-up in the region in which they are diagnosed. Therefore, comprehensive, consecutive records providing almost 100% coverage are available for these patients [13]. Patients included in the study originated from five facilities within the region: Karolinska University Hospital Solna, Karolinska University Hospital Huddinge, Danderyd Hospital, Södersjukhuset and Visby Hospital. Regional ethics committee approval was obtained prior to commencement of the study. As this was a retrospective observational study, no informed patient consent was required. The study was performed in accordance with the ethical principles of the Declaration of Helsinki and in compliance with national laws.

Patient-level data for the Stockholm cohort were obtained from an extended comprehensive retrospective review of patient files identified in the registry [13]. A total of 148 patients with relapsed or refractory CLL were identified, and their files were subject to an in-depth analysis from diagnosis until last treatment line or current line of treatment at last follow-up. Four patients with information related only to their ibrutinib treatment were left out of the comparative analysis, resulting in analysable records for 144 patients. All patients received second-line treatment, and follow-up in subsequent treatment lines was available for patients in their third (*n* = 88), fourth (*n* = 49), fifth (*n* = 25) and sixth and subsequent (*n* = 16) lines of treatment. Patients who moved into further treatment lines after second-line therapy contributed information to the analysis for multiple lines of therapy, resulting in a sample size of 322 treatment lines from 144 patients. Patient characteristics collected at the initiation of each treatment line, reflecting the corresponding baseline status of the patient, are used in the analyses to adjust the comparison for differences versus the ibrutinib cohort. The principle of including the same patient multiple times, each of them with a different point of follow-up, was proposed recently by Hernan et al., who considered this approach more efficient from a statistical standpoint, as long as appropriate adjustment of the usual variance estimator is implemented [15].

The RESONATE cohort (ibrutinib, *n* = 195; ofatumumab, *n* = 196) randomised relapsed/refractory (R/R) patients with CLL to treatment with continuous oral ibrutinib 420 mg once daily until disease progression or intolerable toxic effects or to intravenous ofatumumab for 24 weeks at an initial dose of 300 mg at week 1 followed with a 2000 mg weekly dose for 7 weeks and then every 4 weeks for 16 weeks [10]. All patients initiated treatment between 2012 and 2013. As patients from RESONATE only had one treatment episode of ibrutinib or ofatumumab, only one observation per patient was included in the analysis.

### Statistical analysis

The primary statistical hypothesis of this study was that ibrutinib monotherapy accrued from the RESONATE cohort significantly improves PFS and OS in patients with relapsed or refractory CLL compared with previous standard of care represented by the historical Stockholm cohort.

Initially, a Cox proportional hazards regression model [16] including treatment as the only covariate was developed to estimate the “unadjusted” hazard ratio (HR) of ibrutinib versus previous standard of care (as a measure of relative efficacy/effectiveness for time to event data). Subsequently, to account for observed differences in patient characteristics between the RESONATE cohort and the Stockholm cohort, baseline prognostic factors were added as covariates to the Cox proportional hazards regression model to estimate the “adjusted” HR. In contrast to the (unadjusted) HR from the first model, the adjusted HR estimates for the treatment effect based on this multivariate model are not confounded anymore by differences between cohorts and can be interpreted as reflecting the real relative effect for ibrutinib versus previous standard of care. The list of characteristics included as covariates in the multivariate model was determined by clinical importance and availability in both data sources and included line of therapy, age, gender, Binet stage, Eastern Cooperative Oncology Group (ECOG) performance status and refractory disease. Fluorescence in situ hybridization (FISH) results (del (17p) and/or *TP53* mutation results) were not included in the model due to a lack of such information for most patients from the early years of record keeping: FISH was not introduced in the clinic until later. IGHV mutational status was also not included, as it was not part of the standard-of-care routine analysis in Sweden.

The clustering of observations at treatment line level within patients was controlled for using the robust sandwich estimate for the covariance matrix, making confidence intervals (CI) somewhat more conservative [16–18].

Unadjusted and adjusted HRs (including 95% CI) for the treatments reflecting previous standard of care in the Stockholm cohort relative to ibrutinib in the RESONATE cohort were calculated. HRs for treatment and prognostic covariates from the multivariate models are presented graphically as forest plots, representing point estimates and 95% CIs. PFS was defined as the time between randomisation (when considering the RESONATE cohort) or treatment initiation (when considering the Stockholm cohort) and disease progression or death. OS was defined as the time between randomisation/treatment initiation and death. Patients who were lost to follow-up or did not reach the event of interest were censored at the date of their final assessment. Analysis of efficacy endpoints was conducted on the intention-to-treat population from both cohorts. All statistical analyses were performed using SAS 9.2 (Cary, NC).

## Results

### Patient population

Patient characteristics from the RESONATE cohort and at the initiation of the second or later line of treatment from the Stockholm cohort are shown in Table 1. Whereas the cohorts were comparable with regard to gender distribution, the patients from the Stockholm cohort were older, had higher Binet stage and ECOG scores and included more refractory patients despite having received fewer lines of therapy compared with the RESONATE cohort.

**Table 1:** Patient characteristics for those in the ibrutinib and ofatumumab arms of the RESONATE trial and those of the Stockholm cohort

	Ibrutinib (*N* = 195)	Ofatumumab (*N* = 196)	Stockholm cohort (*N* = 322)^a^
Median age (years)	67	67	72
Age categories (years), no. (%)			
<60	45 (23)	40 (20)	48 (15)
60–<65	32 (16)	35 (18)	24 (7)
65–<70	40 (21)	41 (21)	57 (18)
70–<75	35 (18)	43 (22)	63 (20)
75–<80	29 (15)	21 (11)	61 (19)
≥80	14 (7)	16 (8)	69 (21)
Gender, no. (%)			
Male	129 (66)	137 (70)	220 (68)
Female	66 (34)	59 (30)	102 (32)
BINET stage, no. (%)			
A	36 (19)	35 (18)	39 (12)
B	57 (29)	57 (29)	84 (26)
C	102 (52)	104 (53)	193 (60)
Missing	0	0	6 (2)
ECOG score, no. (%)			
0	79 (41)	80 (41)	75 (23)
1	116 (59)	116 (59)	162 (50)
2	0	0	44 (13)
3	0	0	3 (1)
4	0	0	1 (0.3)
Missing	0	0	37 (11)
Refractory to chemotherapy			
Not refractory	108 (55)	108 (55)	76 (24)
Refractory	87 (45)	88 (45)	246 (76)
Line of therapy			
Second line	35 (18)	53 (27)	144 (45)
Third line	57 (29)	53 (27)	88 (27)
Fourth line	32 (16)	38 (19)	49 (15)
Fifth and subsequent lines	71 (36)	52 (27)	41 (13)

*ECOG* Eastern Cooperative Oncology Group

^a^The total *N* value represents a total patient number of 144 undergoing multiple lines of therapy; i.e., it represents the total number of treatment line analyses, not individual patients

**Fig. 1:**
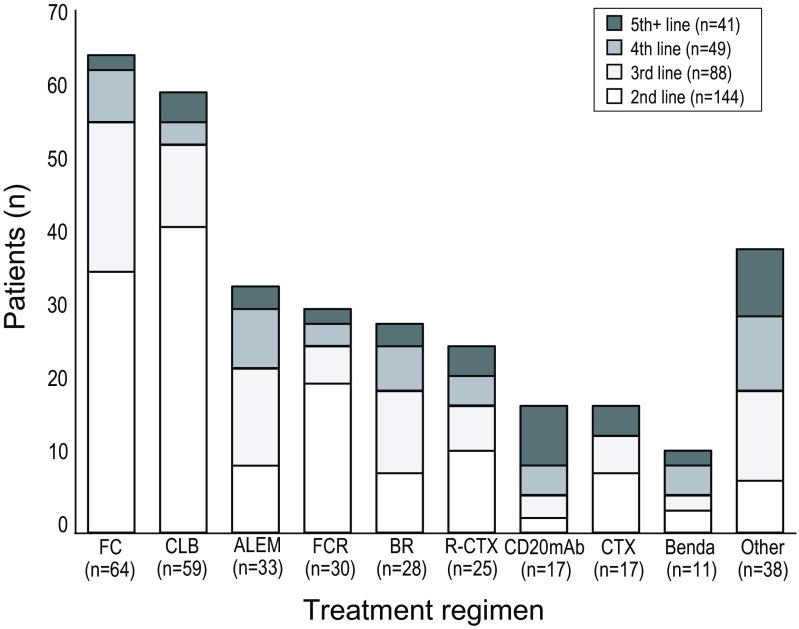
Treatments most frequently used in the Stockholm (previous standard of care) cohort, by line of therapy. *ALEM* alemtuzumab, *Benda* bendamustine, *BR* bendamustine + rituximab, *CD20mAb* (ofatumumab (*n* = 13); rituximab (*n* = 4)) anti-C20 monoclonal antibody, *CLB* chlorambucil, *CTX* chemotherapy (chemotherapy includes various combinations: CVP, CHOP and DHAP), *FC* fludarabine + cyclophosphamide, *FCR* fludarabine + cyclophosphamide + rituximab, *Other* mAb combination therapy, lenalidomide, idelalisib and others, *R-CTX* rituximab + chemotherapy (chemotherapy includes various combinations: CVP, CHOP and DHAP)

The most commonly used drug combinations for each treatment line in the Stockholm cohort (2002–2013) are shown in Fig. 1. Fludarabine-cyclophosphamide (FC) was the most commonly used therapy for all treatment lines taken together (*n* = 64), followed by chlorambucil (CLB) (*n* = 59). In the second line of treatment, CLB was most commonly used therapy (*n* = 41), followed by FC (*n* = 35) and FCR (*n* = 20). Bendamustine was introduced late in the time period studied and was used in the second line in only three patients (*n* = 11 for all treatment lines taken together).

### Efficacy

#### Progression-free survival

**Fig. 2:**
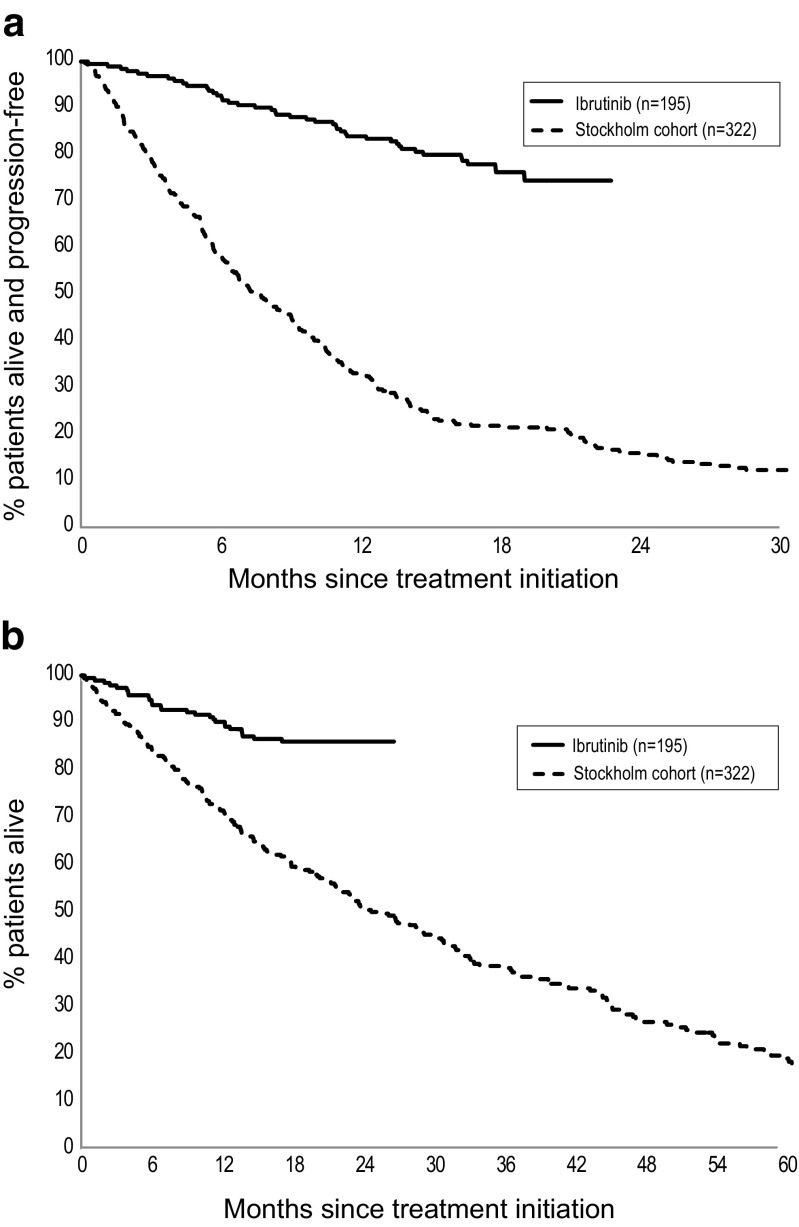
Kaplan-Meier plot for **a** PFS and **b** OS: ibrutinib (IBR) versus Stockholm cohort (previous standard of care)

**Fig. 3:**
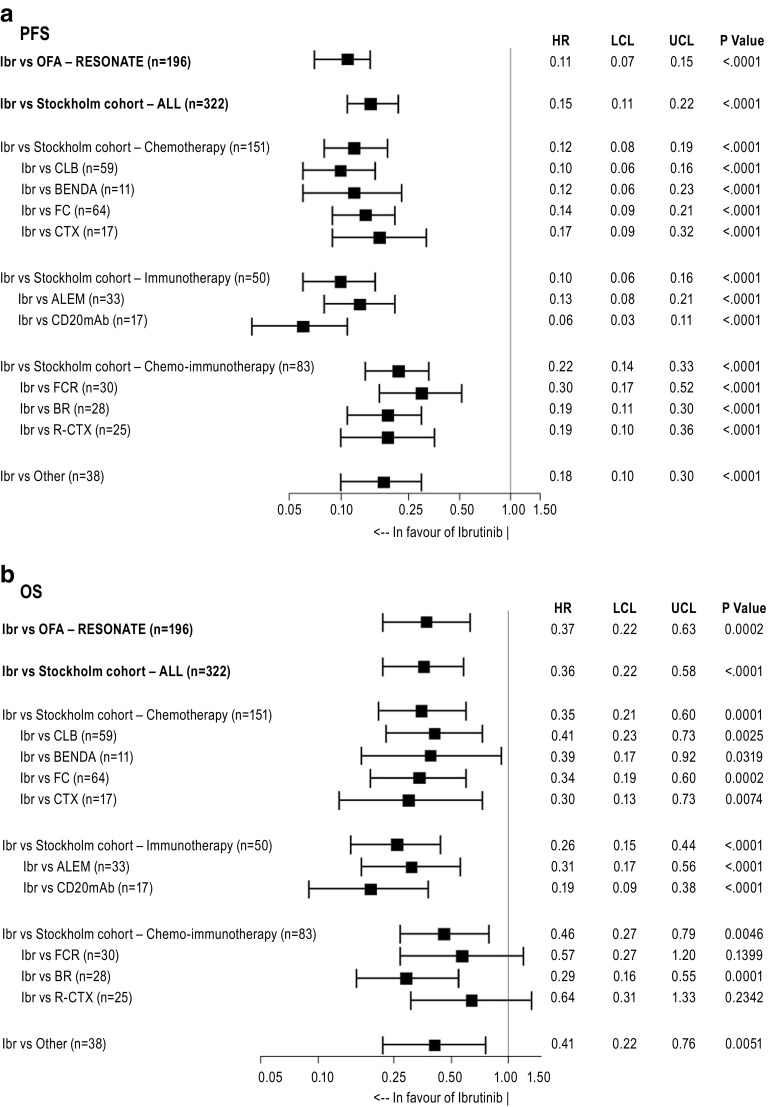
Adjusted HRs (95% CIs) for **a** PFS and **b** OS: ibrutinib (IBR) versus previous standard-of-care regimens as used in the Stockholm cohort (based on multivariate Cox proportional hazards regression). *ALEM* alemtuzumab, *Benda* bendamustine, *BR* bendamustine + rituximab, *CD20mAb* (ofatumumab (*n* = 13); rituximab (*n* = 4)) anti-C20 monoclonal antibody, *CLB* chlorambucil, *CTX* chemotherapy (chemotherapy includes various combinations: CVP, CHOP and DHAP), *FC* fludarabine + cyclophosphamide, *FCR* fludarabine + cyclophosphamide + rituximab, *Ibr* ibrutinib, *OFA* ofatumumab, *Other* mAb combination therapy, lenalidomide, idelalisib and others, *R-CTX* rituximab + chemotherapy (chemotherapy includes various combinations: CVP, CHOP and DHAP), *HR* hazard ratio, *LCL* lower confidence limit, *UCL* upper confidence limit

A Kaplan-Meier plot of PFS for patients treated with ibrutinib versus the Stockholm cohort (previous standard of care) demonstrated a significantly longer PFS for patients on ibrutinib treated in the RESONATE trial compared to previous standard of care as used in routine healthcare (Fig. 2a). The naïve unadjusted HR for ibrutinib versus previous standard of care was 0.16 (95% CI 0.11, 0.22; *p* < 0.0001). When adjusting for differences in observed prognostic risk factors between the cohorts, the HR for ibrutinib versus previous standard of care became 0.15 (95% CI 0.11, 0.22; *p* < 0.001) (Fig. 3a).

Adjusting for differences between cohorts in line of therapy (36% of patients had received five or more lines of therapy in the ibrutinib cohort versus only 13% in the Stockholm cohort—see Table 1) and ECOG status (41% of patients had ECOG 0 in the cohort versus 23% in the Stockholm cohort) had the largest impact on the estimate of the treatment effect on PFS (Table 2, Appendix). As adjustment for both characteristics had an opposite impact (suggesting ibrutinib patients to have more advanced disease based on line of therapy, but less severe based on ECOG), this finally leads to an adjusted HR close to the unadjusted result.

The adjusted PFS HRs for ibrutinib versus individual treatment regimens are depicted in Fig. 3a and ranged between 0.06 (compared with CD20mAb) and 0.30 (compared with FCR) and were statistically significant in all cases. The greatest difference between ibrutinib and other regimens was observed versus immunotherapy alone and versus CLB (both HR = 0.10 [95% CI 0.06, 0.16; *p* < 0.0001]), and the smallest was observed when compared to chemoimmunotherapy treatments (HR = 0.22 [95% CI 0.14, 0.33; *p* < 0.0001]) (Fig. 3a).

The PFS HR for ibrutinib versus the ofatumumab arm from RESONATE (HR = 0.11 [95% CI 0.07, 0.15; *p* < 0.0001]) was similar to the HR versus the Stockholm cohort (HR = 0.15 [95% CI 0.11, 0.22; *p* < 0.0001]).

To explore the potential impact of differences in time periods in which patients were treated, a sensitivity analysis excluding patients from the Stockholm cohort treated before 2012 resulted in a HR similar to the main analysis (HR = 0.15 [0.09; 0.24]).

**Fig. 4:**
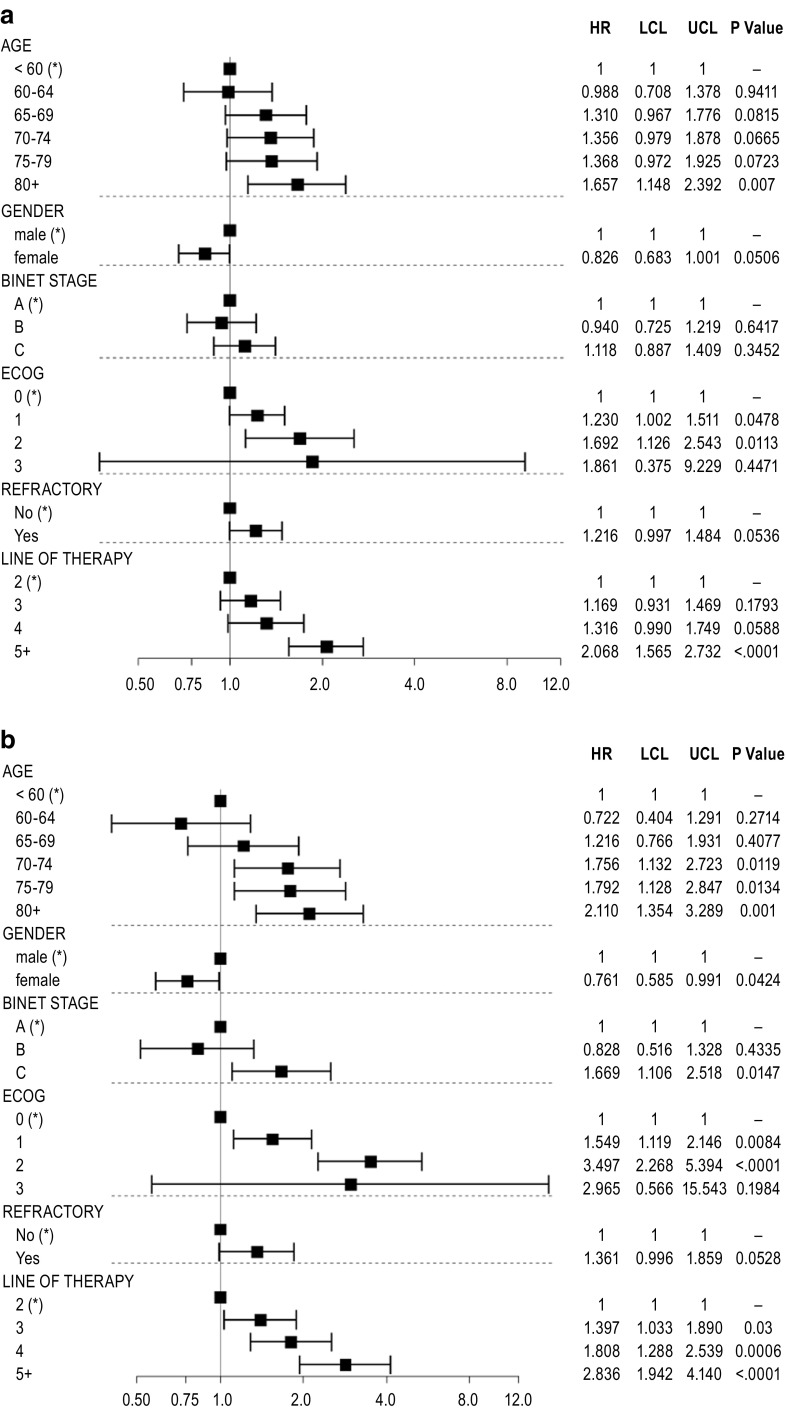
HR estimates for **a** PFS and **b** OS, by level of each baseline characteristic included as covariate in the multivariate Cox proportional hazards regression. Pooled data from the RESONATE and the Stockholm cohorts. **a** *Asterisk*: reference category; HR represents the relative risk by category versus the reference category; estimates above 1 refer to increased risk for progression or death. **b** *Asterisk*: reference category; HR represents the relative risk by category versus the reference category; estimates above 1 refer to increased risk of death. E*COG* Eastern Cooperative Oncology Group, *HR* hazard ratio, *LCL* lower confidence limit, *UCL* upper confidence limit

Figure 4a represents the HRs for all prognostic baseline covariates from the same multivariate Cox model based on the pooled data from RESONATE and Stockholm cohorts, which also generated the adjusted HR for ibrutinib versus previous standard of care reported in Fig. 3a. It illustrates the prognostic value of each baseline characteristic: older age, male gender, Binet C disease stage, poorer ECOG performance status and later line of therapy were all statistically significant independent risk factors for worse outcome on PFS (*p* < 0.05); refractory status was numerically associated with poorer PFS.

The interaction effect of treatment with all baseline characteristics was only significant for age (*p* = 0.025), suggesting the relative treatment effect for ibrutinib in the trial versus previous standard of care to be particularly pronounced in patients between ages 60 and 74 (HR = 0.10), relative to patients below 60 and above 75.

#### Overall survival

A Kaplan-Meier plot of OS for patients treated with ibrutinib versus previous standard of care (Stockholm cohort) also demonstrated a statistically significantly longer OS with ibrutinib (Fig. 2b).

The naïve, unadjusted HR comparing OS for ibrutinib versus previous standard of care was 0.28 (95% CI 0.18, 0.42; *p* < 0.001). After adjustment for differences between cohorts in prognostic risk factors, the HR became 0.36 (95% CI 0.22, 0.58; *p* < 0.001) for ibrutinib versus previous standard of care (Fig. 3b). Similar to PFS, adjusting for differences in line of therapy and ECOG status had the largest impact on the estimate of the treatment effect on OS (Table 2, Appendix).

The greatest OS difference of ibrutinib was seen when compared to immunotherapy-only treatments (HR = 0.26 [95% CI 0.15, 0.44; *p* < 0.0001]) and the smallest difference against chemoimmunotherapy treatments (HR = 0.46 [95% CI 0.27, 0.79; *p* < 0.0046]). The OS HRs for ibrutinib versus individual treatment regimens ranged from 0.19 for CD20mAb to 0.64 for R-CTX and were statistically significant at the 0.05 level in the majority of cases despite low sample sizes (Fig. 3b). The OS HR for ibrutinib versus the ofatumumab arm from RESONATE (HR = 0.37 [95% CI 0.22, 0.63; *p* = 0.0002]) was similar to the HR versus the Stockholm cohort (0.36 [95% CI 0.22, 0.58; *p* < 0.001]).

A sensitivity analysis excluding patients from the Stockholm cohort treated before 2012 provided results consistent with the main analysis (HR = 0.31 [0.15; 0.63]).

Figure 4b shows the prognostic value of all baseline covariates included in the multivariate Cox model based upon data from RESONATE and Stockholm cohorts, to estimate the adjusted OS HRs for ibrutinib versus previous standard of care reported in Fig. 3b. Increasing age, male gender, Binet stage C, poorer ECOG performance status and later line of therapy were all statistically significant independent risk factors for worse outcome with regard to OS at the 5% significance level. Refractory status was numerically associated with poorer OS.

The interaction effect of treatment with baseline characteristics was significant for ECOG (*p* = 0.0001) and Binet stage (*p* = 0.046), suggesting the relative treatment effect regarding OS for ibrutinib versus standard of care to be particularly pronounced in patients with BINET stage A (HR = 0.27) and in patients with ECOG 1 (HR = 0.22).

## Discussion

When evaluating the efficacy of a new class of therapy, previous standard of care and thus the appropriate comparator may differ between countries. There may also exist a wide range of treatment options for a particular disease; then, it may not be financially or logistically practical to compare the new therapy with all available treatment options in a randomised clinical trial setting. In situations where the effectiveness of a new therapy has not yet been directly and proactively assessed in a prospective phase 3 trial, adjusted multivariate analysis of retrospective data may provide a temporary solution. This preliminary comparative information may help assist in healthcare decisions and provide hypothesis-generating results for the next generation of phase 3 clinical trials. Our approach exemplifies a comparison of the efficacy of ibrutinib against previous standard of care in relapsed and refractory patients with CLL by pooling data from a randomised international clinical trial with data from a retrospective observational consecutive cohort of Swedish patients from the Stockholm region with almost complete follow-up and without influence on results from external referrals.

Since some baseline imbalances exist between the cohorts (Table 1), a multivariate Cox proportional hazards regression model was developed which included baseline factors as covariates to adjust for confounding bias related to these differences.

The nature of the Swedish healthcare system means that comprehensive records of both treatment and long-term follow-up of CLL are available for all patients. Additionally, external referrals to the Stockholm region are rare, meaning that all records in the Regional Cancer Registry for the Stockholm region are for patients from one, defined geographical area with minimal external influence, thus minimising selection bias and being a strong representation of the general population. The RESONATE trial took place in multiple countries and adhered to a strict study protocol; the patient population was clearly derived from many geographical regions, which is likely to have introduced heterogeneity in patient characteristics, though both Swedish and RESONATE cohorts were heavily pre-treated. Patients from RESONATE and the historical Stockholm cohort differed in terms of patient characteristics at baseline (Table 1); Swedish patients tended to be older and with higher ECOG scores, and a larger proportion of patients were refractory to prior treatment, making naïve comparisons prone to confounding bias. The adjusted analyses, using multivariate statistical modelling, adjusted for these observed differences between both cohorts. The adjusted HR for OS was less in favour of ibrutinib when compared to results from the “naïve” (unadjusted) comparison, reflecting the fact that the analyses adjust for the higher degree of severity of the Stockholm cohort.

While patients in RESONATE were treated between 2012 and 2015, the Stockholm cohort included patients treated between 2002 and 2013. To explore the potential impact of this difference on the treatment effect estimates, we conducted a sensitivity analysis including only patients in the Stockholm cohort treated 2012 or later. Results for both PFS (HR = 0.15 [0.09; 0.24]) and OS (HR = 0.31 [0.15; 0.63]) were consistent with the main analyses and do not suggest any bias related to the different timeframes for both data sources. Additionally, we recently showed that CLL patients who had received second-line treatment in two time periods (2003–2007 and 2008–2013) displayed a trend of improving PFS over time, but no difference in OS was shown [13]. Taken together, these and other reports on previous generation of salvage therapies [8, 19] suggest that OS for R/R CLL patients was not clearly affected until kinase inhibitors became available.

Although the results obtained from our modelling should be viewed with caution, the data suggest that ibrutinib may provide longer PFS and OS compared with historical standard of care during the time period studied in patients with previously treated CLL. The difference remained intact even when the latest time period in the Stockholm cohort was compared separately. HRs reached statistical significance for most comparisons, even though comparisons versus specific previous generation treatment regimens were based on a small number of patient for a number of treatment regimens.

The adjusted HRs that have been reported in our analysis should be interpreted as estimates for the average treatment effect across the entire patient population included in RESONATE and the historical Stockholm cohort. To what extent the relative treatment effect between ibrutinib versus previous standard of care varied across patients according to their baseline characteristics was explored by additionally including interaction terms for treatment with all baseline characteristics in the statistical models. Results suggest that the effect of ibrutinib on PFS was more pronounced in patients between 60 and 74 compared to younger and older patients. OS effect for ibrutinib versus previous standard of care was significantly higher in Binet stage A patients (compared to stages B and C) and especially in ECOG 1 patients (versus ECOG 0). As none of the patients on ibrutinib had ECOG above 1, it is unclear whether this trend exists in these patients. Importantly, all such subgroup analyses shall be regarded as preliminary and hypothesis-generating only.

In this analysis, it is observed that the relative treatment effects for both PFS and OS of ibrutinib versus the ofatumumab arm within RESONATE and versus the Stockholm cohort are similar. A preliminary interpretation of this finding would be that the outcome observed in the ofatumumab arm within RESONATE can be considered as representative for the outcome of previous standard of care as observed in real clinical practice [10]. The results of the adjusted comparison in this report are in line with the comparison versus the ofatumumab arm within RESONATE study [10]. Additionally, these results are supported by other recent and preliminary reported analyses, where RESONATE trial data were compared with outcome data for R/R CLL patients from other data sources in various ways. A similar statistical modelling approach using patient-level data was applied to compare PFS and OS between ibrutinib monotherapies from RESONATE with bendamustine-rituximab (BR) from the HELIOS trial (comparing ibrutinib plus BR versus BR) [20]. The adjusted HR in that report for ibrutinib versus BR was 0.13 for PFS and 0.45 for OS, which are in line with values reported in our analysis (HR = 0.25 [95% CI 0.14, 0.42] and HR = 0.30 [95% CI 0.16, 0.60], respectively). Doubek et al. compared PFS and OS data drawn from RESONATE with a cohort of R/R CLL patients from academic centres in Czech Republic and reported HRs for PFS (HR = 0.10 [95% CI 0.06, 0.16]) and OS (HR = 0.15 [95% CI 0.08, 0.28]) [21]. Finally, our results are in line with HRs for ibrutinib versus physicians’ choice for PFS (HR = 0.07 CI 0.04; 0.13) and OS (HR = 0.27 CI 0.12; 0.58), based on the Bucher method of adjusted indirect comparison using published results for ibrutinib (RESONATE) and physicians’ choice [8] versus the common comparator ofatumumab [22].

Several limitations should be noted in the interpretation of the results of this study. First, although a wide range of clinically relevant prognostic factors were available to be adjusted for, residual confounding bias cannot be excluded, as is the case in any observational study. In particular, del(17p)/*TP53* mutation, which is a well-known risk factor in CLL, could not be included in the model, due to a lack of such information for most patients from the early years of record keeping. Similarly, IGHV mutational status was also lacking as it was not included in the routine standard-of-care analyses in Sweden. An additional limitation of this report is that time periods when the patients have been treated were different and that duration of follow-up was significantly shorter within RESONATE compared to the previous standard-of-care cohort. However, the PFS and OS associated with ibrutinib were maintained even when restricting the analysis to only patients treated in the same time period (2012–2013). Finally, data from different sources should always be compared with caution.

In conclusion, this study describes a statistical approach which can be used to provide a preliminary comparison between previous real-world treatments and new drugs until comparisons from randomised clinical trials become available.

## Appendix

**Table 2:** HR on OS/PFS for ibrutinib versus Swedish cohort, by incrementally adding covariates to the proportional hazards regression model

	OS	PFS
No covariates	0.28 [0.18; 0.42]	0.16 [0.11; 0.22]
+ Line of therapy	0.18 [0.12; 0.29]	0.12 [0.08; 0.17]
+ ECOG	0.27 [0.17; 0.42]	0.14 [0.10; 0.19]
+ Refractory	0.31 [0.20; 0.49]	0.15 [0.10; 0.21]
+ Age	0.35 [0.22; 0.56]	0.15 [0.11; 0.22]
+ Gender	0.36 [0.22; 0.58]	0.15 [0.11; 0.22]
+ Binet disease stage	0.36 [0.22; 0.58]	0.15 [0.11; 0.22]

## References

[1] Rozovski U, Hazan-Halevy I, Keating MJ, Estrov Z (2014). Personalized medicine in CLL: current status and future perspectives. Cancer Lett.

[2] Shvidel L, Tadmor T, Braester A, Bairey O, Rahimi-Levene N, Herishanu Y, Klepfish A, Ruchlemer R, Berrebi A, Polliack A, Israeli CLLSG (2014). Serum immunoglobulin levels at diagnosis have no prognostic significance in stage A chronic lymphocytic leukemia: a study of 1113 cases from the Israeli CLL Study Group. Eur J Haematol.

[3] Eichhorst B, Dreyling M, Robak T, Montserrat E, Hallek M, Group EGW (2011). Chronic lymphocytic leukemia: ESMO Clinical Practice Guidelines for diagnosis, treatment and follow-up. Ann Oncol.

[4] Hallek M (2015). Chronic lymphocytic leukemia: 2015 update on diagnosis, risk stratification, and treatment. Am J Hematol.

[5] Siegel R, Ma J, Zou Z, Jemal A (2014). Cancer statistics, 2014. CA Cancer J Clin.

[6] NationalCancerInstitute (2016) Surveillance, epidemiology and end results program. 31–3-2016

[7] Thompson PA, Tam CS, O’Brien SM, Wierda WG, Stingo F, Plunkett W, Smith SC, Kantarjian HM, Freireich EJ, Keating MJ (2016). Fludarabine, cyclophosphamide, and rituximab treatment achieves long-term disease-free survival in IGHV-mutated chronic lymphocytic leukemia. Blood.

[8] Österborg A, Udvardy M, Zaritskey A, Andersson PO, Grosicki S, Mazur G, Kaplan P, Steurer M, Schuh A, Montillo M, Kryachok I, Middeke JM, Kulyaba Y, Rekhtman G, Gorczyca M, Daly S, Chang CN, Lisby S, Gupta I (2016) Phase III, randomized study of ofatumumab versus physicians’ choice of therapy and standard versus extended-length ofatumumab in patients with bulky fludarabine-refractory chronic lymphocytic leukemia. Leuk Lymphoma 1–10. doi:10.3109/10428194.2015.1122783 10.3109/10428194.2015.1122783 26784000

[9] Pan Z, Scheerens H, Li SJ, Schultz BE, Sprengeler PA, Burrill LC, Mendonca RV, Sweeney MD, Scott KC, Grothaus PG, Jeffery DA, Spoerke JM, Honigberg LA, Young PR, Dalrymple SA, Palmer JT (2007). Discovery of selective irreversible inhibitors for Bruton’s tyrosine kinase. ChemMedChem.

[10] Byrd JC, Brown JR, O’Brien S, Barrientos JC, Kay NE, Reddy NM, Coutre S, Tam CS, Mulligan SP, Jaeger U, Devereux S, Barr PM, Furman RR, Kipps TJ, Cymbalista F, Pocock C, Thornton P, Caligaris-Cappio F, Robak T, Delgado J, Schuster SJ, Montillo M, Schuh A, de Vos S, Gill D, Bloor A, Dearden C, Moreno C, Jones JJ, Chu AD, Fardis M, McGreivy J, Clow F, James DF, Hillmen P, Investigators R (2014). Ibrutinib versus ofatumumab in previously treated chronic lymphoid leukemia. N Engl J Med.

[11] Byrd JC, Furman RR, Coutre SE, Burger JA, Blum KA, Coleman M, Wierda WG, Jones JA, Zhao W, Heerema NA, Johnson AJ, Shaw Y, Bilotti E, Zhou C, James DF, O’Brien S (2015). Three-year follow-up of treatment-naive and previously treated patients with CLL and SLL receiving single-agent ibrutinib. Blood.

[12] O’Brien S, Jones JA, Coutre SE, Mato AR, Hillmen P, Tam C, Österborg A, Siddiqi T, Thirman MJ, Furman RR, Ilhan O, Keating MJ, Call TG, Brown JR, Stevens-Brogan M, Li Y, Clow F, James DF, Chu AD, Hallek M, Stilgenbauer S (2016). Ibrutinib for patients with relapsed or refractory chronic lymphocytic leukaemia with 17p deletion (RESONATE-17): a phase 2, open-label, multicentre study. Lancet Oncol.

[13] Asklid A, Winqvist M, Eketorp Sylvan S, Mattsson A, Björgvinsson E, Søltoft F, Repits J, Diels J, Österborg A, Hansson L (2016). Outcomes of second-line treatment in chronic lymphocytic leukemia—a population-based study from a well defined geographical region between 2003 and 2013. Leuk Lymphoma.

[14] Forum UC (2016) Ibrutinib for relapsed/refractory CLL: a UK and Ireland analysis of outcomes in 315 patients. Haematologica. doi:10.3324/haematol.2016.147900 10.3324/haematol.2016.147900 PMC5479600 27756834

[15] Hernan MA, Robins JM (2016). Using big data to emulate a target trial when a randomized trial is not available. Am J Epidemiol.

[16] Kleinbaum DG, Klein M (2012) Survival analysis. A self-learning text, third edition. Statistics for biology and health. Springer Science + Business Media. doi:10.1007/978-1-4419-6646-9_1

[17] Lin DY, Wei LJ (1989). The robust inference for the cox proportional hazards model. J Am Stat Assoc.

[18] Robins JM, Hernan MA, Brumback B (2000). Marginal structural models and causal inference in epidemiology. Epidemiology.

[19] Terasawa T, Trikalinos NA, Djulbegovic B, Trikalinos TA (2013). Comparative efficacy of first-line therapies for advanced-stage chronic lymphocytic leukemia: a multiple-treatment meta-analysis. Cancer Treat Rev.

[20] Hillmen P, Fraser G, Jones J, Rule S, O’Brien S, Dilhuydy MS, Jaeger U, Grosicki S, Cymbalista F, Sun S, Ninomoto J, Mahler M, Cheng M, Diels J, Clow F, Salman M, James DF, Howes A, Chanan-Khan A (2015) Comparing single-agent ibrutinib, bendamustine plus rituximab (BR) and ibrutinib plus BR in patients with previously treated chronic lymphocytic leukemia/small lymphocytic lymphoma (CLL/SLL): an indirect comparison of the RESONATE and HELIOS trials. Blood, ASH, 57th Annual Meeting, Abstract 126 (23)

[21] Doubek M, Obrtlikova P, Spacek M, Urbanova R, Diels J, Thilakaratne P, Musingarimi P, Mac Dougall F, Hermans R, Barendse M, Iraqi W, Smolej L (2016). Single-agent ibrutinib vs standard of care for patients with relapsed/refractory (R/R) chronic lymphocytic leukemia (CLL): an adjusted comparison of resonate (TM) with the CLLEAR database. Haematologica.

[22] Tam C, Sorensen S, Sengupta N, Diels J, Van Sanden S (2015). Indirect treatment comparison of ibrutinib versus physician’s choice for treatment of relapsed or refractory chronic lymphocytic leukemia.

